# Evaluation of TTF-1, Napsin A, p40, and p63 in the Subtyping of Non–Small Cell Lung Carcinoma: A Cross-Sectional Study from India 

**DOI:** 10.30699/ijp.2025.2042277.3362

**Published:** 2025-07-01

**Authors:** Surbhi Patel, Deepa Sowkur Anandarama Adiga

**Affiliations:** Department of Pathology, Kasturba Medical College Mangalore, Manipal Academy of Higher Education, Manipal, India

**Keywords:** Lung cancer, Adenocarcinoma, Squamous cell carcinoma, TTF-1, Napsin A, p40, p63

## Abstract

**Background & Objective:**

Subtyping non–small cell lung carcinoma (NSCLC) into adenocarcinoma (ADC) and squamous cell carcinoma (SCC) is crucial for selecting appropriate molecular tests, as driver mutations are often subtype-specific. This study aimed to evaluate the utility of TTF-1, Napsin A, p40, and p63 immunohistochemical (IHC) markers in subtyping NSCLC on small biopsies, with the goal of identifying a minimal marker panel.

**Methods:**

This retrospective, cross-sectional study was conducted at Kasturba Medical College, Mangalore, from January 2014 to December 2020. All NSCLC cases diagnosed during the study period were included. Immunohistochemical expressions of TTF-1, Napsin A, p40, and p63 were evaluated and correlated with morphological findings.

**Results:**

Ninety-five NSCLC cases were included: adenocarcinoma (n = 35), squamous cell carcinoma (n = 57), and NSCLC-not otherwise specified (NOS) (n = 2). IHC reclassification based on marker expression resulted in six ADC cases retyped as SCC and eight SCC cases retyped as ADC. TTF-1 and Napsin A expression were significantly associated with adenocarcinoma (*p* < 0.001), while p40 and p63 expression were significantly associated with SCC (*p* < 0.001).

**Conclusion:**

IHC is essential in overcoming the diagnostic limitations of small biopsy specimens, especially in morphologically heterogeneous tumors. A minimal panel comprising TTF-1 and p40 is sufficient for accurate subtyping of NSCLC and can help preserve tissue for downstream molecular testing.

## Introduction

Lung carcinoma (LC) is one of the most lethal malignancies and remains the leading cause of cancer-related mortality worldwide. In India, it accounts for approximately 5.9% of all cancers, with tobacco smoking being the predominant risk factor in nearly 80% of cases (1). Nevertheless, a substantial proportion of lung cancer cases occur in non-smokers, particularly among females (2, 3).

Lung cancer is broadly classified into small cell lung carcinoma (SCLC) and non–small cell lung carcinoma (NSCLC) based on histopathological characteristics. NSCLC accounts for nearly 85% of all cases and includes adenocarcinoma (ADC), squamous cell carcinoma (SCC), large cell carcinoma (LCC), adenosquamous carcinoma (ASC), and NSCLC-not otherwise specified (NOS), according to the WHO classification (4, 5).

ADC is the most common subtype in India (1) and demonstrates histologic heterogeneity, often expressing pneumocyte- or mucin-related markers. SCC, an epithelial malignancy, is characterized by keratinization, intercellular bridges, or poorly differentiated morphology, with immunohistochemical confirmation through p40 or p63 expression (4–6).

Because approximately 60% of lung cancer cases are diagnosed at advanced stages (III or IV), when surgical resection is typically not feasible, diagnosis often relies on small tissue samples, including core biopsies, fine-needle aspiration (FNA), and cell blocks (4). A multidisciplinary diagnostic approach incorporating light microscopy, IHC, and molecular profiling is essential for accurate classification and clinical decision-making.

The primary immunomarkers used to differentiate NSCLC subtypes include TTF-1 and Napsin A for adenocarcinoma, and CK5/6, p40, and p63 for squamous cell carcinoma (7–9). TTF-1, encoded by the NKX2-1 gene, is a homeodomain-containing transcription factor expressed in pulmonary adenocarcinomas and thyroid neoplasms (7). Napsin A, an aspartic proteinase, is normally found in renal tubules, type II pneumocytes, and ductal epithelium of various glands (8).

p40, an isoform of p63 (ΔNp63), is a highly specific marker for squamous differentiation, while p63, a member of the p53 transcription factor family located on chromosome 3q, is also expressed in normal respiratory epithelium (9). This study was conducted to assess the diagnostic utility of TTF-1, Napsin A, p40, and p63 in accurately subtyping NSCLC on small biopsy specimens, with the objective of identifying a minimal, cost-effective IHC panel suitable for routine clinical practice.

## Materials and Methods

### Ethics and Setting

In this timebound study, lung biopsies obtained from January 2014 to December 2020 from patients with clinically suspected primary lung cancer were included. These samples were received in the Department of Pathology, Kasturba Medical College, Mangalore, and consisted of bronchoscopic biopsies, computed tomography (CT), guided biopsies, ultrasound-guided biopsies, cell blocks, and resected lung specimens. Cases with inadequate biopsy material for immunohistochemistry (IHC) were excluded. The study was approved by the Institutional Ethics Committee (Approval Number: IEC KMC MLR 09-19/395). Since the study was retrospective and based on archival biopsy samples, informed consent was waived.

### Biopsy Review

Following morphological evaluation of hematoxylin and eosin (H&E), stained slides, all cases of non, small cell lung carcinoma (NSCLC) were confirmed and subtyped into adenocarcinoma, squamous cell carcinoma, and NSCLC-not otherwise specified (NOS) based on morphological criteria outlined by the World Health Organization (WHO) and the International Association for the Study of Lung Cancer/American Thoracic Society (IASLC/ATS) classification. Cases diagnosed morphologically as small cell carcinoma and large cell neuroendocrine carcinoma were excluded from the study.

### Immunohistochemistry

IHC was performed on formalin-fixed, paraffin-embedded (FFPE) tissue that had been previously evaluated morphologically. Thin sections were cut from representative blocks onto adhesive-coated slides at the same time as H&E sections to minimize tissue loss. IHC staining was carried out using the following antibodies: TTF-1 (Master Diagnostica, mouse monoclonal, clones 8G7G3/1 and SPT24), Napsin A (Master Diagnostica, mouse monoclonal, clone BS10), p40 (Master Diagnostica, ready-to-use, clone ZR8), and p63 (Master Diagnostica, clone 4A4). All procedures were performed according to the manufacturer's instructions. The secondary antibody used was the Dako REAL EnVision/HRP detection system (labelled polymer, Code K5007), which targets both rabbit and mouse primary antibodies.

Immunohistochemical evaluation for TTF-1, p40, and p63 was based on nuclear staining, while cytoplasmic staining was considered for Napsin A. Particular attention was given to excluding entrapped pneumocytes and macrophages, which may show background positivity for TTF-1 and Napsin A, to ensure accurate interpretation of tumor-specific staining.

### Statistical Analysis

Data were analyzed using the Statistical Package for the Social Sciences (SPSS) version 25.0. Descriptive statistics such as mean, proportion, and standard deviation were used to summarize the findings. Clinical and demographic variables in this study were categorical. Age groups were divided into four categories with 10-year intervals for comparison. The Chi-square test and Fisher’s exact test were used to evaluate associations between categorical variables. A p-value of less than 0.05 was considered statistically significant.

## Results

During the study period, a total of 560 cases of lung carcinoma were diagnosed on biopsy. Of these, 70 cases were diagnosed as small cell carcinoma, and 490 were classified as non–small cell lung carcinoma (NSCLC). Among these, 95 consecutive NSCLC cases that met the inclusion and exclusion criteria were included in the study. This group consisted of 92 small biopsies, including 55 bronchoscopic biopsies, 36 CT-guided biopsies, and one ultrasound-guided biopsy. In addition, two lobectomy specimens and one cell block were also analyzed.

The majority of patients in the study were between 61 and 70 years of age, accounting for 37% of the total cases. A significant male predominance was noted, with a male-to-female ratio of 4.5:1. A history of current or previous tobacco smoking was present in 70% of the patients.

Based on morphology alone, the frequency of each subtype is shown in Table 2.

Adenocarcinoma

**Fig 1:**
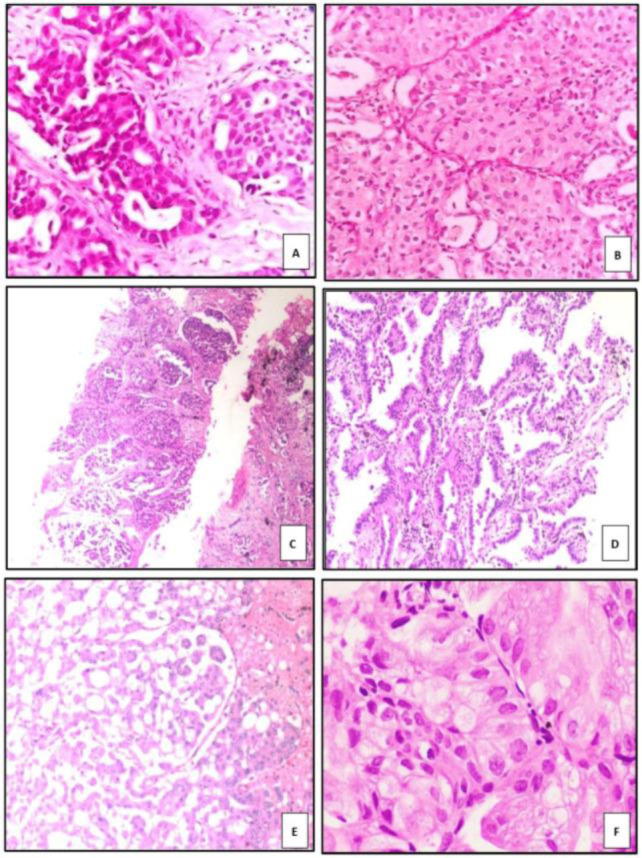
Patterns of ADC: A) acinar pattern B) solid pattern C) cribriform pattern D) lepidic pattern, non mucinous type E) micropapillary pattern F) lepidic pattern, mucinous type.

Morphologically, most adenocarcinoma cases exhibited a combination of acinar (77.1%) and solid (85.7%) arrangements in varying proportions. The tumor cells displayed vacuolated to eosinophilic cytoplasm in 51.4% of cases, vesicular nuclei in 51.4%, and prominent nucleoli in 82.9%, all of which were statistically significant (*p* < 0.0001) (Fig. 1).

**Table 1:** Clinicopathological characteristics of Adenocarcinoma and Squamous cell carcinoma

Category		ADC (Count)	ADC (%)	SCC (Count)	SCC (%)
Age (in years)	50 and below	10	28.6%	10	17.85%
	51-60	8	22.9%	14	25%
	61-70	11	31.4%	20	35.7%
	Above 70	6	17.1%	12	21.42%
Sex	Female	11	31.42%	4	7.14%
	Male	24	68.57%	52	92.8%
Smoking	Present	21	60%	42	75%
	Absent	8	23%	7	12.5%
	Not applicable	6	17.14%	7	12.5%
Location	Central	6	17.14%	42	75%
	Peripheral	29	82.57%	14	25%

**Table 2:** Frequency of distribution of subtype of Non‑Small Cell Lung Carcinoma (NSCLC) based on morphology.

Histologic description	Cases
Adenocarcinoma	35
Squamous cell carcinoma	57
NSCLC with adenosquamous morphology	1
NSCLC – not otherwise specified	2
Total	95

Squamous Cell Carcinoma

Most squamous cell carcinoma cases showed a nest-like arrangement of tumor cells (100%), with eosinophilic cytoplasm suggestive of individual cell keratinization observed in 76.6% of cases. Intercellular bridges were present in 75.4%, and hyperchromatic nuclei were identified in 92.9% of cases, all demonstrating high statistical significance (*p* < 0.0001). Keratin pearl formation was seen in 78.9% of cases.

The tumor in the case with adenosquamous carcinoma (ASC) morphology revealed solid nests of keratinized cells with keratin pearls, along with a distinct component of tumor cells arranged in a glandular pattern.

Two cases were morphologically unclassifiable as either adenocarcinoma or squamous cell carcinoma. Both cases exhibited solid sheets of tumor cells lacking definitive glandular or squamous differentiation. One of these cases demonstrated marked pleomorphism with bizarre nuclear features suggestive of sarcomatoid morphology.

**Immunohistochemical Analysis**

The immunohistochemical expression profiles of TTF-1, Napsin A, p40, and p63 in adenocarcinoma are summarized in Table 3. Two adenocarcinoma cases exhibited tumor cells with abundant intracellular mucin (Fig. 1F). These cases were negative for TTF-1, Napsin A, p40, p63, and CK20, but showed positive cytoplasmic staining for CK7.

**Fig. 2:**
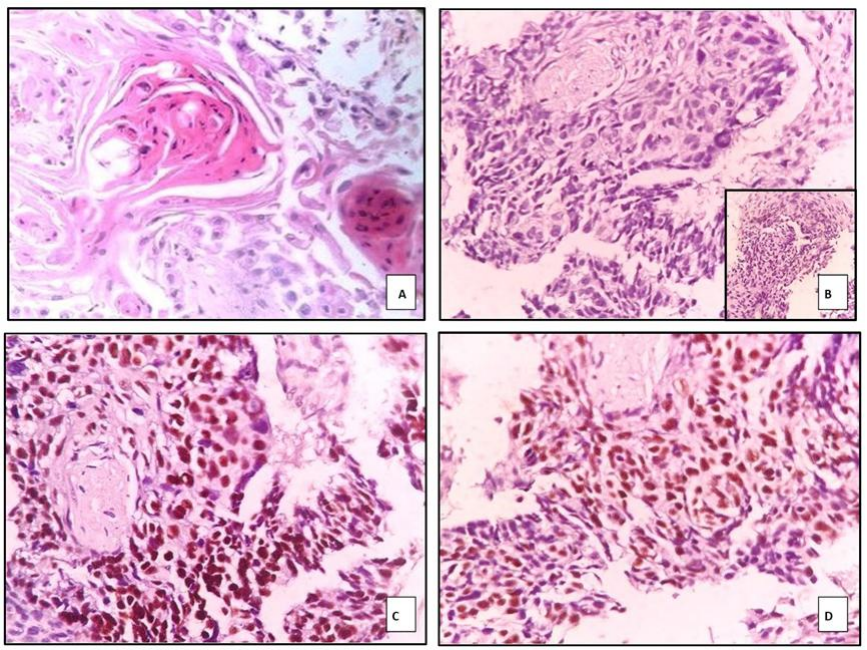
Photograph of keratinized SCC in A) H&E, 400X, Negative marker expression for TTF-1 and Napsin A (inset) in B) with positive expression for p40 in C) and p63 in D

Among the 57 cases morphologically diagnosed as squamous cell carcinoma (SCC), 49 cases showed strong and diffuse nuclear positivity for p40, and 48 cases showed similar nuclear positivity for p63 (Fig. 2). The remaining eight cases, which were negative for both p40 and p63, exhibited nuclear positivity for TTF-1 and cytoplasmic staining for Napsin A. Based on this immunoprofile, these eight cases were reclassified as adenocarcinoma. Details of the immunohistochemical findings in SCC cases are presented in Table 4.

**Table 3:** Immunohistochemical marker expression in morphologically diagnosed adenocarcinoma

Adenocarcinoma	TTF-1	Napsin A	p40	p63	
Positive	24	22	6	3 (focal & weak)	3 (strong & diffuse)
Negative	11	13	29	29	
Total	35				

**Table 4:** p40, p63, TTF-1 and Napsin A immunohistochemical expression in morphologically diagnosed Squamous cell Carcinoma (SCC)

Squamous cell carcinoma	p40	p63	TTF-1	Napsin A
Positive	49	48	8	8
Negative	8	9	49	49
Total	57			

**Table 5:** Immunohistochemical expression of TTF-1, Napsin A, p40 and p63 in morphologically diagnosed Non‑Small Cell Lung Carcinoma- Not otherwise Specified (NSCLC NOS)

NSCLC‑NOS	p40	p63	TTF-1	Napsin A
Positive	1	1	1	1
Negative	1	1	1	1
Total	2			

Morphologically, two cases were initially diagnosed as non–small cell lung carcinoma–not otherwise specified (NSCLC-NOS). After immunohistochemical analysis, one case was reclassified as adenocarcinoma (ADC) and the other as squamous cell carcinoma (SCC) with sarcomatoid features (Table 5).

One biopsy revealed malignant tumor nests exhibiting both squamous and glandular differentiation. Immunohistochemical staining demonstrated marker expression for both squamous and adenocarcinoma components in two distinct cell populations, supporting a diagnosis of adenosquamous carcinoma.

Adenocarcinoma

TTF-1 expression was observed in 33 of 35 adenocarcinoma cases, while 2 cases were negative. Among the positive cases, 31 demonstrated strong and diffuse nuclear positivity, and 2 cases showed focal and weak nuclear staining. The *p*-value for TTF-1 expression in adenocarcinoma was 0.000, indicating high statistical significance.

**Fig. 3:**
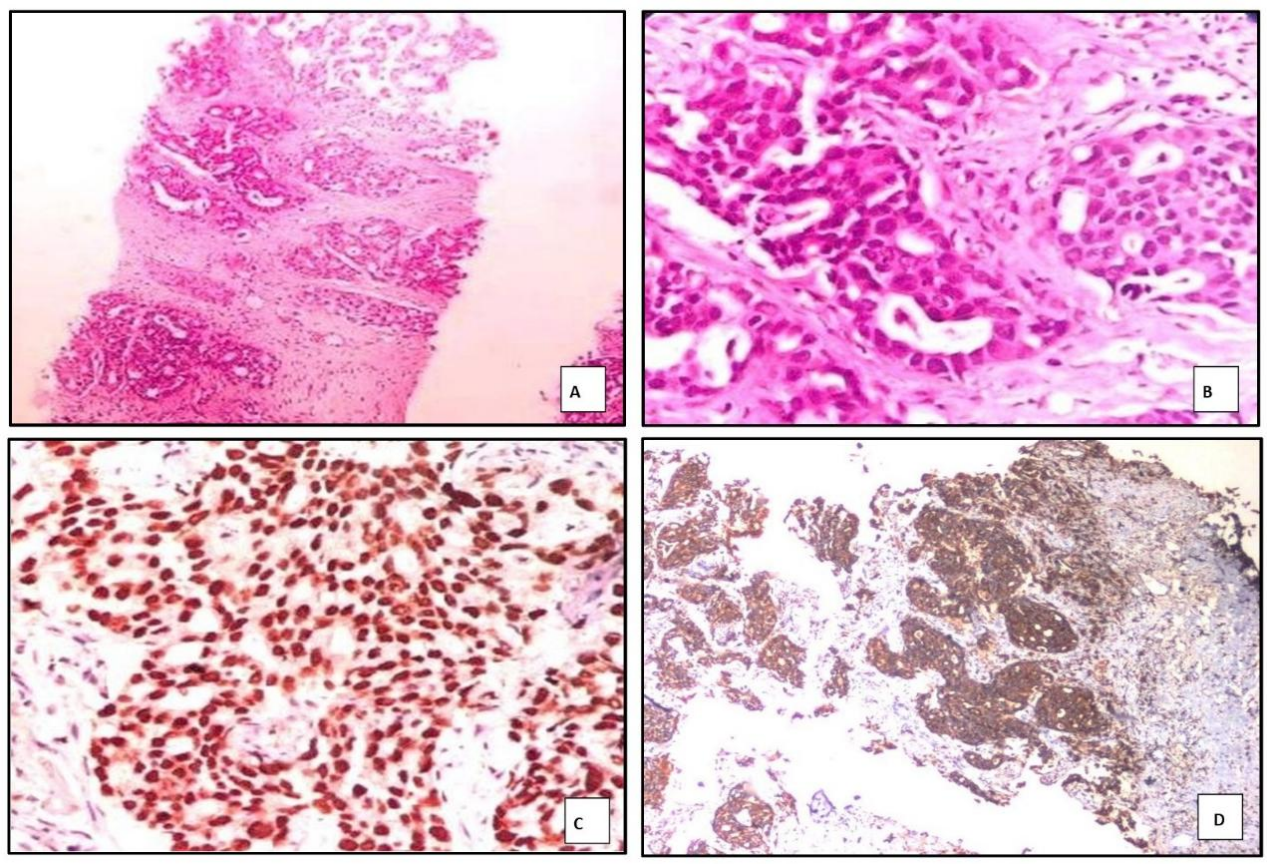
Photomicrograph showing ADC of lung with tumour cells in acinar pattern in A) H&E, 100X, and B)H&E 400X, with C) strong, diffuse, nuclear TTF-1 positivity and D) strong, diffuse, cytoplasmic Napsin A positivity

Napsin A was expressed in 31 out of 35 cases of adenocarcinoma. Among these, 29 cases exhibited strong and diffuse cytoplasmic staining, whereas 2 showed focal and weak positivity. Four cases were negative for Napsin A expression. The *p*-value for Napsin A expression in adenocarcinoma was also 0.000, which is highly significant (Fig. 3).

p63 was expressed in 3 of 35 adenocarcinoma cases, all of which showed only focal and weak nuclear staining. All adenocarcinoma cases were negative for p40. 

**Fig. 4:**
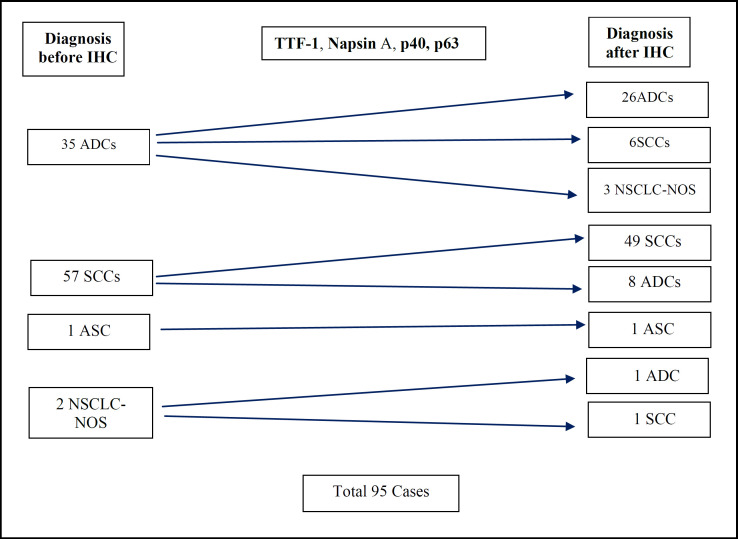
Line diagram depicting the redistribution of subtypes of non‑small cell carcinoma of lung based on diagnosis, before and after immunohistochemistry.

**Fig. 5:**
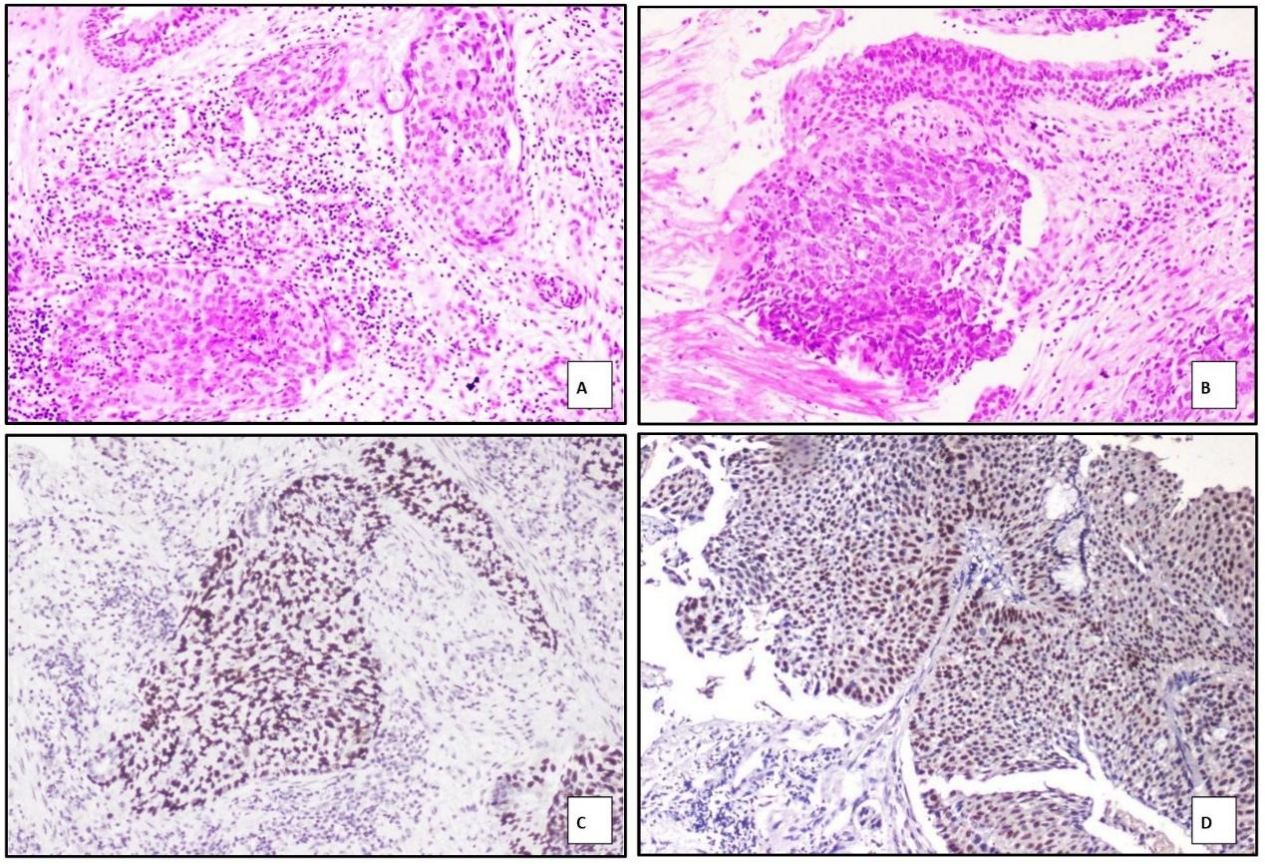
Photomicrograph showing nests of tumor cells with a basaloid morphology mimicking small cell carcinoma. H&E 400X in A) and B). Strong nuclear positivity for p40 in C) and D).

## Discussion

Driver mutations in lung cancer (LC) play a fundamental role in tumorigenesis. EGFR, KRAS, and ALK mutations are considered prototypical driver mutations in LC (8). EGFR mutations occur more frequently among females and non-smokers (9,10). Oncogenic alterations such as mutations in EGFR, KRAS, BRAF, and ERBB2; translocations involving ALK, ROS1, and RET; and amplifications of MET and FGFR1 in both adenocarcinoma (ADC) and squamous cell carcinoma (SCC) have provided new therapeutic avenues and enabled recognition of molecular subsets that can predict treatment response (11,12). However, identifying these alterations requires adequate tissue, which must be preserved during diagnostic and immunohistochemical (IHC) procedures. This necessitates strategies to minimize tissue depletion, hence the rationale for identifying a minimal IHC panel.

Most NSCLC patients present with advanced-stage disease and are treated with targeted therapies based on driver mutations detected. Accordingly, accurate subtyping of NSCLC into SCC and ADC has become essential for guiding treatment decisions (13). Because many of these patients are eligible only for small biopsies or cytology samples, careful utilization of limited tissue is critical for both subtyping and mutation testing. A minimal IHC panel becomes a practical necessity in conserving tissue for predictive biomarker analysis (11–13).

In our study, lung cancer was more prevalent among males and in individuals aged 61–70 years, consistent with previous literature (3,5,7,14). ADC cases displayed varied histologic patterns, which are clinically relevant as they determined prognosis and guided treatment strategies (16). Mucin staining was not performed due to its low specificity in non-mucinous ADC, its cross-reactivity with SCC tumor cell inclusions, and the limited tumor volume in many biopsies. Furthermore, mucin staining was omitted to preserve tissue for molecular testing. Notably, lepidic predominant patterns in early-stage ADC are associated with favorable prognosis (17,18).

In our study, we identified one resected ADC case exhibiting spread through air spaces (STAS) and a high proportion of micropapillary patterns. Terada et al. found STAS in 46 of 76 patients with papillary and micropapillary features and noted worse outcomes in STAS-positive ADCs (23,24).

TTF-1 expression was noted in 94.28% of ADCs in our study, a highly significant finding (p < 0.005) and consistent with other reports (1,25–27). Napsin A was expressed in 88.57% of ADCs (p < 0.005), aligning with similar studies (8,28,29). p63 was expressed in 8.57% (3/35) of ADC cases in a weak and focal manner, consistent with findings by Argon et al. and Bir et al. (32,33). These data highlight the diagnostic pitfall of p63, emphasizing the importance of interpreting p63 in the context of TTF-1 expression. All ADC cases were negative for p40, confirming its specificity for SCC.

Among the 56 SCC cases, classic histopathologic features were noted, including keratin pearls (78.94%), intercellular bridges (75.43%), and individual cell keratinization (76.68%), all statistically significant (p < 0.005), consistent with SCC morphology (34). One case demonstrated a basaloid pattern mimicking small cell carcinoma but was strongly positive for p40 and p63, supporting a diagnosis of basaloid SCC. Moro-Sibilot et al. reported that basaloid patterns correlate with poor prognosis (35), and the WHO 2020 classification recommends using squamous markers to distinguish such variants from mimics (34).

All SCC cases in our study were positive for p40, reaffirming its high sensitivity and specificity for squamous differentiation, as supported by other studies (35–38). One study by Affandi et al. showed lower positivity (77.1%), possibly due to smaller sample size (37). p63 was positive in 98.21% (55/56) of SCC cases, again in agreement with previous reports (35,37,38). All SCC cases were negative for TTF-1 and Napsin A. While some studies report TTF-1 positivity in SCC, this has been attributed to the use of the less specific SPT24 clone (39). In our study, the 8G7G3/1 clone was used in most cases, and even in five cases stained with SPT24, no TTF-1 positivity was seen in SCC.

Given its higher specificity, p40 is preferred over p63 for confirming squamous differentiation. One morphologically ambiguous case showed glandular and squamous components and was diagnosed as adenosquamous carcinoma (ASC). Although the diagnosis of ASC requires resected specimens for confirmation based on component proportions (34,40), suggesting its possibility in biopsy helps inform prognosis, as ASC is typically more aggressive (15,22).

Morphological heterogeneity and small biopsy limitations often complicate NSCLC subtyping. Eleven of 35 cases initially categorized morphologically as ADC were later reclassified after IHC. Six were redefined as SCC and five as NSCLC-NOS. These cases had a solid nested appearance with eosinophilic or clear cytoplasm and lacked keratin pearls. This reinforces the limitations of morphology alone in subtyping NSCLC and supports the role of IHC (14,42–45). Among the NSCLC-NOS cases, further subtyping was not possible even after IHC. These tumors warrant broad cytokeratin testing and exclusion of metastasis, provided molecular testing is not compromised.

IHC also helped reclassify eight morphologically diagnosed SCC cases as ADC, based on marker profiles. These cases had solid patterns and artifactually formed intercellular bridges. Coupling morphology with IHC is thus essential for accurate classification.

Two ADC cases in our study exhibited invasive mucinous adenocarcinoma (IMA) morphology. Both were negative for TTF-1, Napsin A, and CK20 but positive for CK7, consistent with known IMA features (34,46). TTF-1 positivity in IMA varies; some studies report only 43% positivity (44,45). These tumors frequently harbor KRAS mutations and are more common in non-smokers. Both our IMA cases involved elderly, never-smoking females with advanced disease. Recognizing this subtype, even on biopsy, is important due to its poor prognosis.

### Limitations

A key limitation of this study was the unavailability of corresponding resection specimens to confirm the subtyping of NSCLC. This was primarily due to the advanced, unresectable stage at which most patients presented with the disease. Nonetheless, the immunohistochemical markers utilized in this study have been well-established and validated in the literature as reliable diagnostic tools for identifying primary adenocarcinoma and squamous differentiation on resected specimens (46, 47).

## Conclusion

Our findings underscore that subtyping NSCLC based on morphology alone can be challenging due to tumor heterogeneity and the limited tissue available in small biopsies. Immunohistochemistry serves as a crucial adjunct to morphology in accurately subtyping NSCLC, thereby reducing the proportion of cases classified as NSCLC-NOS. Among the tested markers, TTF-1 demonstrated high sensitivity and specificity for primary lung adenocarcinomas, while p40 was more specific than p63 for identifying squamous differentiation. Employing a minimal immune-histochemical panel comprising TTF-1 and p40 enables reliable subtyping in most NSCLC cases, thus conserving tissue for essential molecular testing of targetable driver mutations.

## References

[1] Singh N, Agrawal S, Jiwnani S, Khosla D, Malik PS, Mohan A et al (2021). Lung Cancer in India. J Thorac Oncol.

[2] Shankar S, Thanasekaran V, Dhanasekar T, Duvooru P (2014). Clinicopathological and immunohistochemical profile of non-small cell lung carcinoma in a tertiary care medical centre in South India. Lung India.

[3] Darling HS, Viswanath S, Singh R, Ranjan S, Pathi N, Rathore A (2020). A clinico-epidemiological, pathological, and molecular study of lung cancer in Northwestern India. J Cancer Res Ther.

[4] Travis WD, Brambilla E, Burke AP, Marx A, Nicholson AG (2015). Introduction to The 2015 World Health Organization Classification of Tumors of the Lung, Pleura, Thymus, and Heart. J Thorac Oncol.

[5] Noronha V, Dikshit R, Raut N, Joshi A, Pramesh CS, George K (2012). Epidemiology of lung cancer in India: focus on the differences between non-smokers and smokers: a single-centre experience. Indian J Cancer.

[6] Toh CK, Gao F, Lim WT, Leong SS, Fong KW, Yap SP (2006). Never-smokers with lung cancer: epidemiologic evidence of a distinct disease entity. J Clin Oncol.

[7] van Zyl A, Schubert PT, Koegelenberg CFN (2019). The utility of TTF-1, napsin A, CK5 and p63 staining in the sub-classification of non-small cell carcinoma of the lung. Cytopathology.

[8] Turner BM, Cagle PT, Sainz IM, Fukuoka J, Shen SS, Jagirdar J (2-12). Napsin A, a new marker for lung adenocarcinoma, is complementary and more sensitive and specific than thyroid transcription factor 1 in the differential diagnosis of primary pulmonary carcinoma: evaluation of 1674 cases by tissue microarray. Arch Pathol Lab Med.

[9] Yatabe Y, Kerr KM, Utomo A, Rajadurai P, Tran VK, Du X (2015). EGFR mutation testing practices within the Asia Pacific region: results of a multicenter diagnostic survey. J Thorac Oncol.

[10] Wakejima R, Inamura K, Ninomiya H, Nagano H, Mun M, Okumura S (2020). Mucinous lung adenocarcinoma, particularly referring to EGFR-mutated mucinous adenocarcinoma. Pathol Int.

[11] Shames DS, Wistuba II (2014). The evolving genomic classification of lung cancer. J Pathol.

[12] Li T, Kung HJ, Mack PC, Gandara DR (2013). Genotyping and genomic profiling of non-small-cell lung cancer: implications for current and future therapies. J Clin Oncol.

[13] Nicholson AG, Gonzalez D, Shah P, Pynegar MJ, Deshmukh M, Rice A (2010). Refining the diagnosis and EGFR status of non-small cell lung carcinoma in biopsy and cytologic material, using a panel of mucin staining, TTF-1, cytokeratin 5/6, and P63, and EGFR mutation analysis. J Thorac Oncol.

[14] Walia R, Jain D, Madan K, Sharma MC, Mathur SR, Mohan A (2017). p40 & thyroid transcription factor-1 immunohistochemistry: A useful panel to characterize non-small cell lung carcinoma-not otherwise specified (NSCLC-NOS) category. Indian J Med Res.

[15] Wei JW, Tafe LJ, Linnik YA, Vaickus LJ, Tomita N, Hassanpour S (2019). Pathologist-level classification of histologic patterns on resected lung adenocarcinoma slides with deep neural networks. Sci Rep.

[16] Micke P, Botling J, Mattsson JSM, Planck M, Tran L, Vidarsdottir H (2019). Mucin staining is of limited value in addition to basic immunohistochemical analyses in the diagnostics of non-small cell lung cancer. Sci Rep.

[17] Yoshizawa A, Motoi N, Riely GJ, Sima CS, Gerald WL, Kris MG (2011). Impact of proposed IASLC/ATS/ERS classification of lung adenocarcinoma: prognostic subgroups and implications for further revision of staging based on analysis of 514 stage I cases. Mod Pathol.

[18] Kadota K, Villena-Vargas J, Yoshizawa A, Motoi N, Sima CS, Riely GJ (2014). Prognostic significance of adenocarcinoma in situ, minimally invasive adenocarcinoma, and nonmucinous lepidic predominant invasive adenocarcinoma of the lung in patients with stage I disease. Am J Surg Pathol.

[19] Ito M, Miyata Y, Yoshiya T, Tsutani Y, Mimura T, Murakami S (2017). Second predominant subtype predicts outcomes of intermediate-malignant invasive lung adenocarcinoma. Eur J Cardiothorac Surg.

[20] Kim M, Chung YS, Kim KA, Shim HS (2019). Prognostic factors of acinar- or papillary-predominant adenocarcinoma of the lung. Lung Cancer.

[21] Sica G, Yoshizawa A, Sima CS, Azzoli CG, Downey RJ, Rusch VW (2010). A grading system of lung adenocarcinomas based on histologic pattern is predictive of disease recurrence in stage I tumors. Am J Surg Pathol.

[22] Tsubokawa N, Mimae T, Miyata Y, Sasada S, Yoshiya T, Kushitani K (2016). Prognostic significance of vascular invasion in intermediate-grade subtype of lung adenocarcinoma. Jpn J Clin Oncol.

[23] Toki MI, Harrington K, Syrigos KN (2020). The role of spread through air spaces (STAS) in lung adenocarcinoma prognosis and therapeutic decision making. Lung Cancer.

[24] Terada Y, Takahashi T, Morita S, Kashiwabara K, Nagayama K, Nitadori JI (2019). Spread through air spaces is an independent predictor of recurrence in stage III (N2) lung adenocarcinoma. Interact Cardiovasc Thorac Surg.

[25] Stenhouse G, Fyfe N, King G, Chapman A, Kerr KM (2004). Thyroid transcription factor 1 in pulmonary adenocarcinoma. J Clin Pathol.

[26] Li W, Niehaus AG, O'Neill SS (2020). Immunohistochemistry Profile Predicts EGFR Mutation Status in Lung Adenocarcinoma. Int J Surg Pathol.

[27] Wang Z, Li M, Huang Y, Ma L, Zhu H, Kong L (2018). Clinical and radiological characteristics of central pulmonary adenocarcinoma: a comparison with central squamous cell carcinoma and small cell lung cancer and the impact on treatment response. Onco Targets Ther.

[28] Mohammed Salama ME (2020). Role of Napsin A and Survivin Immunohistochemical Expression in Bronchogenic Adenocarcinoma. Asian Pac J Cancer Prev.

[29] Jin L, Liu Y, Wang X, Qi X (2018). Immunohistochemical analysis and comparison of napsin A, TTF1, SPA and CK7 expression in primary lung adenocarcinoma. Biotech Histochem.

[30] Whithaus K, Fukuoka J, Prihoda TJ, Jagirdar J (2012). Evaluation of napsin A, cytokeratin 5/6, p63, and thyroid transcription factor 1 in adenocarcinoma versus squamous cell carcinoma of the lung. Arch Pathol Lab Med.

[31] Choy B, Findeis-Hosey JJ, Li F, McMahon LA, Yang Q, Xu H (2013). High frequency of coexpression of maspin with p63 and p53 in squamous cell carcinoma but not in adenocarcinoma of the lung. Int J Clin Exp Pathol.

[32] Argon A, Nart D, Veral A (2015). The Value of Cytokeratin 5/6, p63 and Thyroid Transcription Factor-1 in Adenocarcinoma, Squamous Cell Carcinoma and Non-Small-Cell Lung Cancer of the Lung. Turk Patoloji Derg.

[33] Bir F, Aksoy Altınboga A, Satiroglu Tufan NL, Kaya S, Baser S, Yaren A (2014). Potential utility of p63 expression in differential diagnosis of non-small-cell lung carcinoma and its effect on prognosis of the disease. Med Sci Monit.

[34] Nicholson AG, Tsao MS, Beasley MB, Borczuk AC, Brambilla E, Cooper WA (2022). The 2021 WHO Classification of Lung Tumors: Impact of Advances Since 2015. J Thorac Oncol.

[35] Bishop JA, Teruya-Feldstein J, Westra WH, Pelosi G, Travis WD, Rekhtman N (2012). p40 (ΔNp63) is superior to p63 for the diagnosis of pulmonary squamous cell carcinoma. Mod Pathol.

[36] Nakajima N, Yoshizawa A, Moriyoshi K, Sonobe M, Menju T, Sumiyoshi S (2019). P40 expression in small cell lung cancer: The presence of p40-positive cells does not always indicate squamous differentiation. Thorac Cancer.

[37] Affandi KA, Tizen NMS, Mustangin M, Zin RRMRM (2018). p40 Immunohistochemistry Is an Excellent Marker in Primary Lung Squamous Cell Carcinoma. J Pathol Transl Med.

[38] Nonaka D (2012). A study of ΔNp63 expression in lung non-small cell carcinomas. Am J Surg Pathol.

[39] Rekhtman N, Ang DC, Sima CS, Travis WD, Moreira AL (2011). Immunohistochemical algorithm for differentiation of lung adenocarcinoma and squamous cell carcinoma based on large series of whole-tissue sections with validation in small specimens. Mod Pathol.

[40] Kumar R, Singh GK, Singh P (2020). Coughing up: "Adenosquamous carcinoma lung with unusual initial presentation as an ulceroproliferative growth" - case report and review of literature. J Cancer Res Ther.

[41] Moro-Sibilot D, Lantuejoul S, Diab S, Moulai N, Aubert A, Timsit JF (2008). Lung carcinomas with a basaloid pattern: a study of 90 cases focusing on their poor prognosis. Eur Respir J.

[42] Sun F, Wang P, Zheng Y, Jia W, Liu F, Xiao W (2018). Diagnosis, clinicopathological characteristics and prognosis of pulmonary mucinous adenocarcinoma. Oncol Lett.

[43] Righi L, Vavalà T, Rapa I, Vatrano S, Giorcelli J, Rossi G (2014). Impact of non-small-cell lung cancer-not otherwise specified immunophenotyping on treatment outcome. J Thorac Oncol.

[44] Paulk A, Tavora F, Burke A (2018). Pulmonary mucinous adenocarcinomas: a clinicopathologic series with emphasis on the prognostic significance of spread through alveolar spaces, and presence of solid growth component. Surg Exp Pathol.

[45] Gow CH, Hsieh MS, Liu YN, Lee YH, Shih JY (2021). Clinicopathological Features and Survival Outcomes of Primary Pulmonary Invasive Mucinous Adenocarcinoma. Cancers (Basel).

[46] Mukhopadhyay S, Katzenstein AL (2011). Subclassification of non-small cell lung carcinomas lacking morphologic differentiation on biopsy specimens: Utility of an immunohistochemical panel containing TTF-1, napsin A, p63, and CK5/6. Am J Surg Pathol.

[47] Pelosi G, Fabbri A, Bianchi F, Maisonneuve P, Rossi G, Barbareschi M (2012). ΔNp63 (p40) and thyroid transcription factor-1 immunoreactivity on small biopsies or cellblocks for typing non-small cell lung cancer: a novel two-hit, sparing-material approach. J Thorac Oncol.

